# Deep Learning Body Region Classification of MRI and CT Examinations

**DOI:** 10.1007/s10278-022-00767-9

**Published:** 2023-03-09

**Authors:** Philippe Raffy, Jean-François Pambrun, Ashish Kumar, David Dubois, Jay Waldron Patti, Robyn Alexandra Cairns, Ryan Young

**Affiliations:** 1 Present Address: Clairity, Austin, TX USA; 2 Enterprise Imaging Solutions, Change Healthcare, 10711 Cambie Road, Richmond, BC V6X 3G5 Canada; 3 Present Address: Accenture, San Francisco, CA USA; 4 Mecklenburg Radiology Associates, Charlotte, NC USA; 5 University of British Columbia, Vancouver, BC Canada; 6 Present Address: Allen Institute for AI, Seattle, WA USA

**Keywords:** Anatomy, Classification, Deep learning, Machine learning, Medical imaging, CT, MRI

## Abstract

This study demonstrates the high performance of deep learning in identification of body regions covering the entire human body from magnetic resonance (MR) and computed tomography (CT) axial images across diverse acquisition protocols and modality manufacturers. Pixel-based analysis of anatomy contained in image sets can provide accurate anatomic labeling. For this purpose, a convolutional neural network (CNN)–based classifier was developed to identify body regions in CT and MRI studies. Seventeen CT (18 MRI) body regions covering the entire human body were defined for the classification task. Three retrospective datasets were built for the AI model training, validation, and testing, with a balanced distribution of studies per body region. The test datasets originated from a different healthcare network than the train and validation datasets. Sensitivity and specificity of the classifier was evaluated for patient age, patient sex, institution, scanner manufacturer, contrast, slice thickness, MRI sequence, and CT kernel. The data included a retrospective cohort of 2891 anonymized CT cases (training, 1804 studies; validation, 602 studies; test, 485 studies) and 3339 anonymized MRI cases (training, 1911 studies; validation, 636 studies; test, 792 studies). Twenty-seven institutions from primary care hospitals, community hospitals, and imaging centers contributed to the test datasets. The data included cases of all sexes in equal proportions and subjects aged from 18 years old to + 90 years old. Image-level weighted sensitivity of 92.5% (92.1–92.8) for CT and 92.3% (92.0–92.5) for MRI and weighted specificity of 99.4% (99.4–99.5) for CT and 99.2% (99.1–99.2) for MRI were achieved. Deep learning models can classify CT and MR images by body region including lower and upper extremities with high accuracy.

**Supplementary Information:**

The online version contains supplementary material available at 10.1007/s10278-022-00767-9.

## Introduction

Accurate anatomic region labeling of medical images is required for classification of body parts included in medical imaging studies. Body part study labels contain key information used to search, sort, transfer, and display medical imaging datasets across clinical and research healthcare systems [1]. Unfortunately, with the increase in multisystem imaging techniques and consolidation or sharing of Picture Archiving and Communication System (PACS) datasets, currently implemented body part image labeling methods can fall short resulting in incomplete selection and presentation of important relevant imaging studies in clinical viewers. Additionally, the increased demand for automated image-based post-processing workflows, automated selection of studies for clinical AI analysis, and automated anatomical-based study selection for development of AI research datasets has accelerated the need for improved efficiency and reliability of anatomical image labeling techniques.

Ideally, labeling of cross-sectional medical images should accurately reflect the anatomy contained in the individual image and identify all body regions included in a study. Currently, for MR and CT, applied body region labels at the image, series, and study level are often limited to one predominant body region (e.g., chest or abdomen) and do not indicate other body regions included in the scan or do not define a body region (e.g., PET CT or whole-body MR). Furthermore, the lack of standardization of anatomic labels between institutions and human data entry errors both contribute to unreliable anatomy-based labeling of imaging studies. These labeling limitations can adversely affect imaging workflows. They have the potential to adversely affect image interpretation if they result in automated hanging protocols failing to display all information relevant to accurate image interpretation or fail to correctly select data for automated post-processing, including clinical AI workflows. The limitations result in the use of manual search strategies for procurement of anatomically based dataset for AI research, which are prohibitive to rapid developments.

We describe two pixel-based models to automatically identify 17 body regions in CT (CT model) and 18 body regions in MRI studies (MRI model). Our approach improves on some of the limitations of previous attempts to tackle this classification problem using supervised and unsupervised deep learning techniques. Previous publications have shown accuracy results ranging from 72 to 92% [2–6] but were limited to CT only. Several other limitations of the previous publications include the study design (lack of independent test dataset, repeat studies, lack of information about image inclusion/exclusion criteria), the neural network architecture (legacy neural nets), the size of the dataset (< 1700 total patients, ≤ 100,000 total images), the number of body region classes (≤ 12, no upper extremities), and the extent of clinical protocols covered (mostly thin slice CT acquisitions, no contrast medium). To our knowledge, this represent the first description of an anatomical classifier that can reach state-of-the-art accuracy or weighted sensitivity greater than 90% in CT and MRI across a large spectrum of body regions, patient demographics, patient comorbidities, and clinical imaging protocols.

## Materials and Methods

### Study Design

The performance of each AI model (CT, MRI) was evaluated in a retrospective standalone study using manually defined ground truth data. Unless specified otherwise, the two models leveraged most of the same processing pipeline including the neural network architecture. For each modality, three datasets were collected for AI model training and evaluation: training, validation, test (holdout set). The selection of body regions was established with the goal of covering the entire human body. Seventeen CT (18 MRI) body regions were considered for the classification task: head, neck, chest, breast (MRI only), abdomen, pelvis/hip, thigh/upper leg, knee, calf/lower leg, foot, shoulder, humerus/arm, elbow, forearm, hand, spine cervical, spine thoracic, spine lumbar. Non-contrast scans were collected for all body regions and complemented with MRI and CT contrast datasets for the head, neck, chest, and abdomen. The studies with contrast included a spectrum of contrast phases, such as portal venous phase, arterial phase, and delayed phase imaging.

### Data

Both training/validation and testing data came from private sources of pre-existing cases through master agreements with external partners detailing de-identification, scope of use, and selection criteria. As this is a low privacy risk retrospective study using de-identified data from a large cohort of patients for which contact information is not available, the IRB waived authorization requirements as per HIPAA privacy rule. Partners arranged for the collection and de-identification of data to comply with HIPAA requirements, and the industry authors controlled the data. The de-identification schema followed the one used in the Cancer Imaging Archive initiative [7].

The data used for training and validation originated from a large cohort of 63,699 de-identified studies (CT, 28,211 patients; MRI, 35,488 patients) from one healthcare system and its affiliates (University of Wisconsin Health). Patients underwent contrast or non-contrast imaging between 1997 and 2020 (median: 2017) and came from a tertiary care hospital as well as a smaller affiliated hospital and several outpatient imaging centers. A well-controlled selection process was used to build the training and validation datasets. In the first phase, the data was selected from this large pool to ensure a representative distribution of body regions. In the second phase, several iterations of active learning, which consist of inferring results on unlabeled data, computing an uncertainty score, and labeling the top 200 most uncertain studies, [8, 9] were applied to the large cohort of unlabeled data until the accuracy goal was reached. This step was added to enrich the datasets with more complex anatomical cases.

The test datasets were collected from a different source, e.g., United Point Health system (UPH) and its affiliates. Because of the over-representation of datasets acquired with a GE scanner in the training and validation datasets, the instruction was given to focus on collecting primarily test datasets acquired on scanner vendors other than GE scanner. The same attention to collecting a balanced representation of all the body parts was given. The CT and MR imaging data from 3003 patients scanned between 2016 and 2020 (median: 2020) was collected and served as a pool of data to build the test datasets. These patients came from primary care hospitals, critical care hospitals, and imaging centers.

This all-comers study was designed with the intent to be as inclusive as possible and clinically relevant (Tables 2 and 3, and Supplemental Materials – Inclusion and exclusion criteria). All selected patients were included in the study irrespective of their demographic (e.g., ethnicity) or comorbidities. Due to the under-representation of pediatric cases, test datasets were only composed of adult patients. The same inclusive approach was followed for acquisition protocols, orientations (supine, prone, lateral), CT kernels, and MRI sequences. The only exclusions were applied to protocols where the structural information was insufficient such as flow protocols and MRI elastography (Supplemental Materials – Inclusion and exclusion criteria). Models were trained on 2D transversal slices. Axial images with an angle up to 45° were included to cover oblique acquisitions. Multiplanar CT reformatted series such as coronal and sagittal series as well as series used to aid planning of the acquisition such as scout, calibration, and quality control series were excluded. Similarly, all post-processed series such as perfusion maps, reformat, 3D reconstruction, secondary capture, CAD, CINE, and movies were not considered. Image exclusion criteria were image stored in a format 8 bits or lower, image with multiple channels (RGB or other not grayscale), no pixel data, very limited amount of pixel data (< 1000 pixels), and no compatible codec (anything not JPEG lossless, Raw RLE).

### Ground Truth

**Fig. 1:**
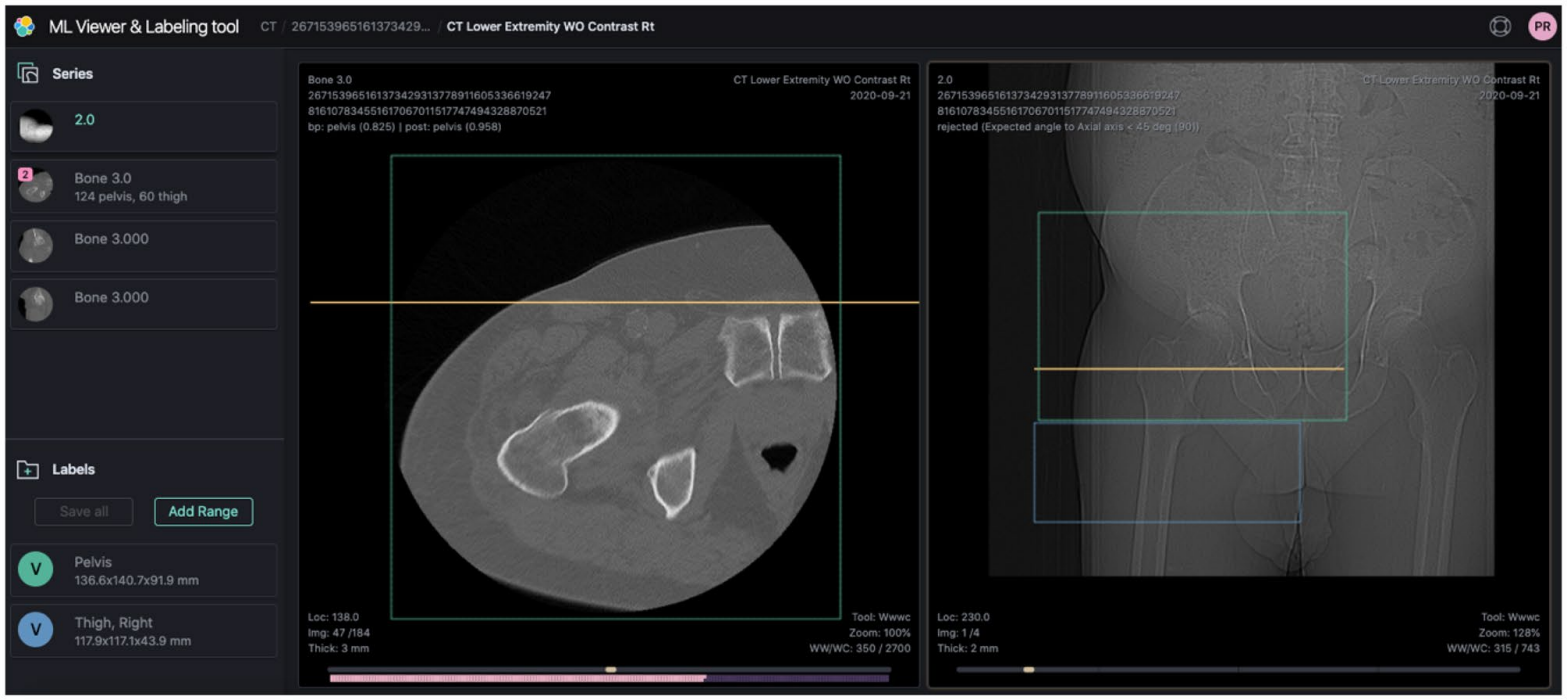
Labeling can be done on any cross-section series (axial, sagittal, coronal, oblique). In this example, a rectangle is first drawn on the axial plane to indicate the pelvis area and then extended on the coronal viewport to the lesser trochanter. This creates a thin 3D bounding box which can be easily manipulated in all dimensions to cover the whole anatomy. The 3D bounding box label is automatically carried over to all other series within the same frame of reference using the patient coordinate system. With this technique, hundreds of images can be labeled in few seconds with a handful of clicks. If needed, AI body region image inference results can be made available with a color code associated with each anatomical class (see bottom color bar). For this image, the AI prediction indicates a pelvis (pink) with a confidence level of 0.825. Lower in the scan, thigh images (purple) are also correctly predicted

﻿The ground truth was labeled based on clearly defined anatomical landmarks (Table 1) using an in-house annotation software (Fig. 1). To avoid the effect of inter-reader variability, all the datasets were labeled by one image annotator with a long-time experience of developing CAD solutions (PR). Pediatric patients under 10 years old were reviewed and edited independently by an experienced pediatric radiologist (RAC) but were left out of the final analysis because of insufficient number of samples. To evaluate the accuracy and consistency of the ground truth, an independent review of a random subset of the labeled dataset was conducted by an experienced radiologist (JWP) and the results compared with the initial annotator. Consistent with peer review practices [10], 2.5% of the labeled data was reviewed with an equal number of studies assigned for all body regions.

**Table 1:** Anatomical landmarks for all 18 body region classes

**Body region**	**Anatomical landmarks (top)**	**Anatomical landmarks (bottom)**
Abdomen	Diaphragm/lung base	Bifurcation of the aorta
Breast (MRI only)	Skin surface of upper breast at chest wall	Skin surface of lower breast at chest wall
Calf/lower leg	Proximal 6th of tibia	Distal third of calf
Chest	Lung apex	Lung base
Elbow	Distal 6th of humerus	Proximal 6th of radius
Foot	Distal third of calf	Bottom of the foot
Forearm	Proximal 6th of radius	Distal 6th of radius
Hand	Distal 6th of radius	Tip of finger
Head	Top of head	Bottom of skull base (foramen magnum)
Humerus/arm	Proximal 6th of humerus	Distal 6th of humerus
Knee	Distal 6th of femur	Proximal 6th of tibia
Neck	Skull base (foramen magnum)	Lung apex
Pelvis and hip	Aortic bifurcation	Lesser trochanter (hip), inferior extent of pubis symphysis (pelvis)
Shoulder	Top of acromioclavicular (AC) joint	Proximal 6th of humerus
Spine cervical	Tip of odontoid	Bottom of T1
Spine thoracic	Top of T1	Bottom of T12
Spine lumbar	T11	Mid sacrum (S2, S3)
Thigh/upper leg	Lesser trochanter	Distal 6th of femur

### Data Partitions

﻿Test and validation datasets were stratified on patient ID so that one patient could not be present in both datasets. Labeled datasets were organized according to the main body region and sorted according to study size. A 75/25 split between the training and validation patient datasets was performed for each body region. To estimate the size of the test datasets, a strict survey study sampling model [11] was used with the assumptions of a model at least 90% accurate and a 95% confidence interval with a 10% relative error. Based on this model, it was determined that at least 7600 images per body region for CT and at least 3600 images per body region for MRI were needed.

### Model

**Fig. 2:**
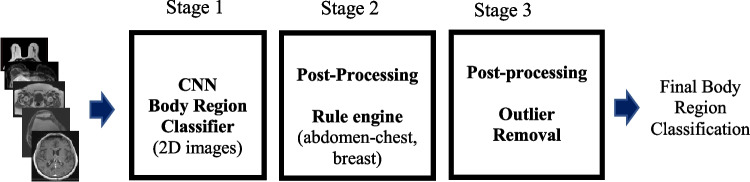
Body region processing steps. To accommodate the model’s input, several pre-processing steps were applied at the image level. First, pixel values were clipped to fit the interval [mean − 4*std, mean + 4*std], where mean and std correspond to the mean value and standard deviation of pixels in the image. Second, pixel values were normalized using the following transform (pixel value − mean)/(2*std) to have input values in the range −1 to 1 to match pretrained models’ requirements. Third, the grayscale images were converted to RGB images by copying pixel data from first channel to the second and third channels. Last, each image was resized with zero padding to fit the model’s required input size of 224 × 224 pixels. Following the 2D CNN image classifier, two rules were applied at the series level to merge the abdomen-chest class to either the abdomen or chest class depending on which body region was predominant. In the absence of chest and abdomen predictions, an abdomen-chest prediction was classified as the abdomen. A second rule was classified as the breast when at least 50% of the images in the series were classified as such. This is to eliminate spurious measurements in noisy breast acquisitions. In the last stage of post-processing, we first applied a routine at the series level to remove outlier labels. We then applied a moving average filter with a window size of three pixels to smooth out results so a continuity in classified labels could be observed throughout the series

The classification task is composed of multiple stages that are detailed in Fig. 2. As a first step, we used the standard ResNet50V2 model [12] in a multi-class framework. Following the 2D CNN classifier, a few post-processing steps at the series level were applied. First, a rule engine merged the abdomen-chest class to both the abdomen and chest class and classified an entire series as breast if at least 50% of the images in the series were classified as such. Last, a smoothing step was applied to remove labels inconsistent with those in the immediate vicinity, increasing the consistency of the labels and decreasing noise.

**Fig. 3:**
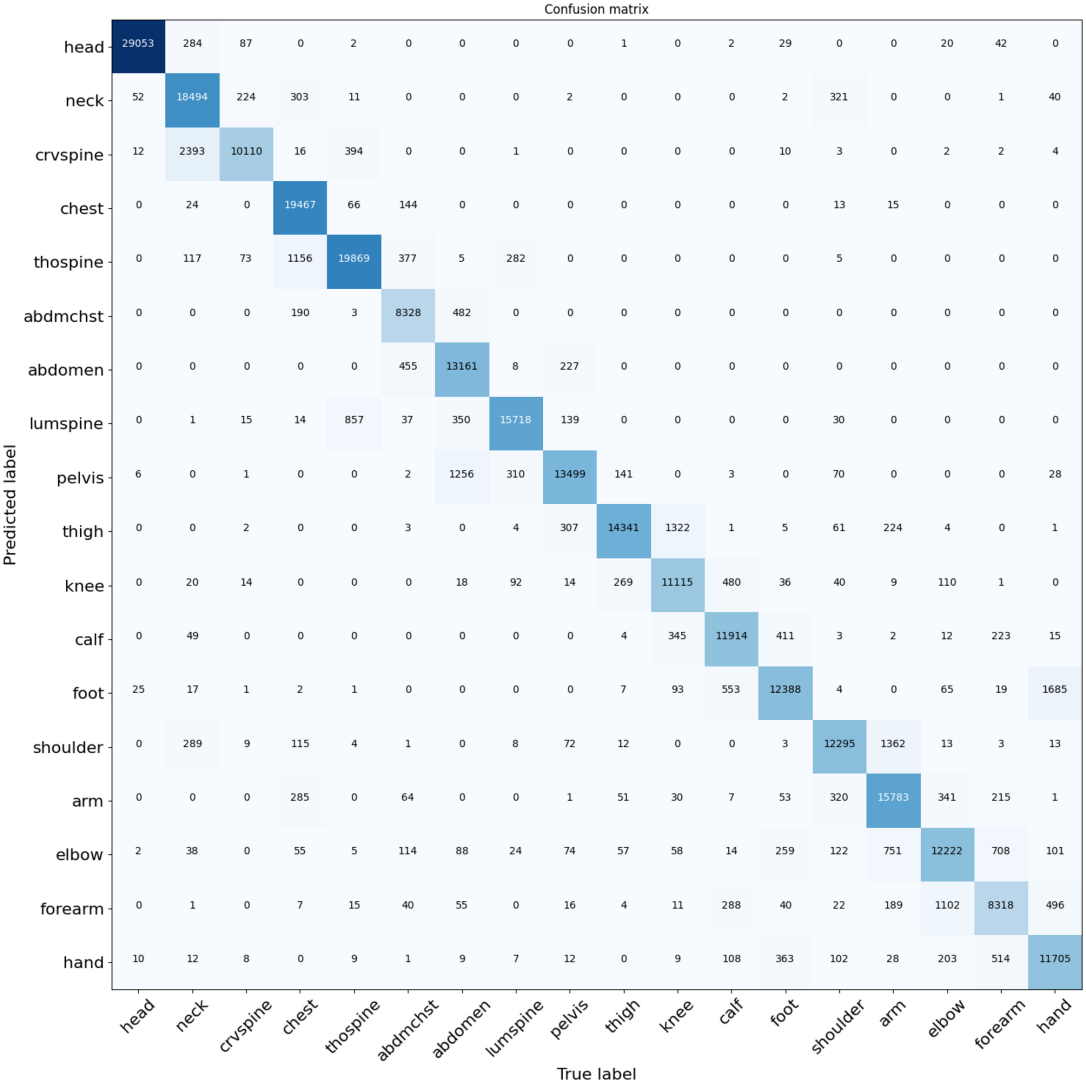
Confusion matrix for the 262,326 images in the CT test database with a threshold of 0.5. Rows represent the predictions, and columns represent the ground truth

### Hardware and Framework

The ML experimentations took place in a containerized cloud environment using TensorFlow 2.3.0. The docker images were built with GPU support. Argo was used to manage the execution of the data ingestion, training, evaluation, and reporting workflows, while MLflow was used to manage the experimental results and generated artifacts such as the model checkpoints, reports, and figures. An 8 CORE CPU, 30 GB RAM, NVIDIA V100 GPU cloud instance was used for training both models. An 8 CORE CPU, 30 GB RAM, NVIDIA T4 GPU cloud instance was used for running the TensorFlow Serving inference engine for both models. All the pre-processing and post-processing steps were written in Python, while the ingestion control, results aggregation, and dispatch were implemented using node.js.

### Training

We enriched our dataset by applying spatial deformations to a random set of images in each training epoch. These transforms include rotation within an angle of ± π/10, translation, and shear with a maximum of 10% in image size in both directions, scaling with a maximum of 20% in image size in both directions and bilinear interpolation. The transformations were applied using built in TensorFlow library functions. We used the transfer learning approach and model weights from pretrained Resnet50V2 model developed for vision benchmark ImageNet dataset. The training hyperparameters are listed in Supplemental Materials – Training Parameters. The loss function used is categorical cross entropy which is well-suited for the multi-class case. We trained each model with all available slices in each series.

### Evaluation

﻿﻿We applied the models to the test datasets and evaluated them by computing the average weighted values of the following performance metrics: F1 score, sensitivity, and specificity. Details are provided in the supplemental materials as to the choice of metrics. The choice is also based on information found in [13]. Results were derived by body region, institution, patient demographics, and acquisition parameters (manufacturer, contrast, CT kernel, slice thickness, sequence type). Performance metrics and their corresponding confidence intervals were determined using the spatial aware bootstrap resampling method [14]. The image sampling procedure ensured that no image slices were closer than 10 mm based on slice position and slice thickness information. This is consistent with the 7.5-mm sampling approach reported in [15]. This spatial aware random sampling was performed at the series level to reduce the impact of strongly correlated images and provide more realistic statistical results. To reduce the inter-series correlation, one series per study (randomly selected at each sampling iteration) in the subsampled dataset was kept. The correlation and correlation significance between the model’s accuracy and each confounding factor was assessed using Cramer’s V and Pearson's chi-squared statistical test.

## Results

### Data

**Fig. 4:**
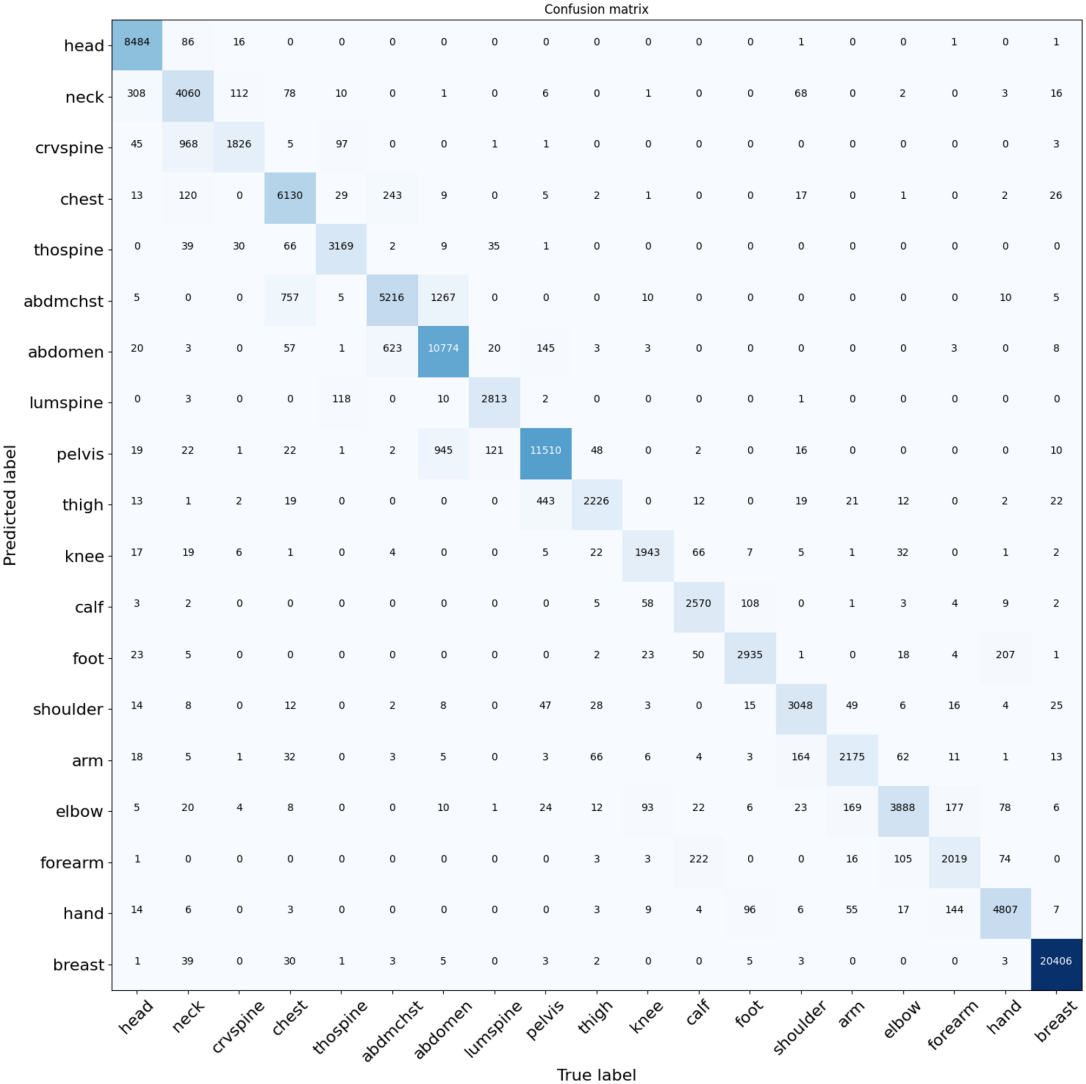
Confusion matrix for the 118,829 images in the MRI test database with a threshold of 0.5. Rows represent the predictions, and columns represent the ground truth

The data consisted of 2891 CT cases (training, 1804 studies; validation, 602 studies; test, 485 studies) and 3339 MRI cases (training, 1911 studies; validation, 636 studies; test, 792 studies). Flowcharts in the Supplemental Materials Inclusion and Exclusion Criteria (Figs. 1–4) show the distribution of images after the different stages of series and image exclusion criteria. The evaluation of the ground truth revealed a total of 4 labeling errors out of 1455 CT and MRI-labeled studies, which represents an error rate of 0.3%.

Distributions of images and results by confounding factors for the test sets can be found in Tables 2 and 3. Twenty-seven institutions contributed to each CT and MRI test dataset. For CT, 56% of datasets came from primary care hospitals and 44% from critical access hospitals and imaging centers, while for MRI, 55% of the datasets came from primary care hospitals. Sex parity was respected for the CT dataset. A slight over-representation of female sex was noticed for the MRI dataset (56.1%). The age coverage ranged from 18 years old to + 90 years, roughly following the distribution of imaging tests in US Healthcare Systems [16]. Compared to the development datasets (Supplemental Materials – Distribution Development Datasets), the test datasets differed in some key areas. For CT, acquisitions mostly originated from Siemens and non-GE scanners (87.6%, + 80.1%) with a larger proportion of older adults ≥ 65 years (45.6%, + 9.3%), intermediate slice thickness (2 mm < slice thickness < 5 mm) (54.8%, + 46.3%), and non-contrast imaging (76.5%, + 9.1%). For MRI, acquisitions mostly originated from Siemens and non-GE scanner (83.5%, + 69%), a larger proportion of older adults (31.7%, + 11.7%), cases with slice thickness > 2 mm and < 5 mm (68.5%, + 20.3%), and non-contrast imaging (84.0%, + 6.4%).

**Table 2:** CT image performance metrics by confounding factors. *n* = number of studies (*series). The *p*-value for the median chi-square is provided to determine if a significant difference in accuracy is found for each confounding factor

	**Category**	**n (%)**	**F1 (95% CI)**	**Sensitivity (95% CI)**	**Specificity (95% CI)**	***p*-value**
**Institution**	Primary care Hospital	272 (56.1)	92.1 (91.6–92.5)	92.1 (91.7–92.5)	99.5 (99.5–99.5)	0.119
	Community Hospital	111 (22.9)	91.5 (90.9–92.2)	91.5 (90.8–92.2)	99.4 (99.3–99.4)	
	Imaging center	102 (21.0)	93.9 (93.4–94.4)	94.0 (93.6–94.6)	97.1 (96.8–97.4)	
**Age**	18–44 years	103 (21.2)	91.0 (90.3–91.8	91.0 (90.3–91.8)	99.4 (99.4–99.5)	6.7e − 7
	45–64 years	161 (33.2)	91.4 (90.9–92.0)	91.5 (91.0–92.1)	99.4 (99.3–99.4)	
	≥ 65 years	221 (45.6)	93.7 (93.3–94.1)	93.7 (93.3–94.1)	99.5 (99.5–94.5)	
**Sex**	Female	245 (50.5)	91.8 (91.4–92.3)	91.8 (91.4–92.3)	99.4 (99.4–99.4)	0.004
	Male	240 (49.5)	93.0 (92.6–93.4)	93.1 (92.7–93.5)	99.5 (99.5–99.5)	
**Manufacturer**	Canon	2 (0.4)	NA	NA	NA	0.003
	GE	62 (12.4)	93.4 (92.7–94.2)	93.6 (92.7–94.4)	99.5 (99.4–99.5)	
	Hitachi	4 (0.8)	NA	NA	NA	
	Philips	23 (4.6)	92.9 (91.4–94.4)	93.0 (91.6–94.5)	99.3 (99.1–99.5)	
	Siemens	343 (68.5)	92.3 (91.9–92.6)	92.3 (92.0–92.7)	99.3 (99.3–99.4)	
	Toshiba	50 (10.0)	91.5 (90.5–92.5)	91.3 (90.2–92.4)	99.4 (99.3–99.5)	
	Vital images	17 (3.4)	100.00 (91.4–100.0)	100.0 (90.0–100.0)	100.0 (98.1–100.0)	
Contrast*	With contrast	305 (26.1)	94.9 (94.4–95.3)	94.9 (94.5–95.4)	98.8 (98.7–99.0)	7.0e − 8
	Without contrast	865 (73.9)	91.5 (91.1–91.8)	91.4 (91.1–91.8)	99.5 (99.5–99.5)	
Slice thickness*	≤ 2 mm	464 (39.7)	92.7 (92.3–93.1)	92.7 (92.3–93.1)	99.6 (99.5–99.6)	7.7e − 9
	> 2 mm and < 5 mm	641 (54.8)	91.8 (91.4–92.3)	91.9 (91.5–92.3)	99.1 (99.1–99.2)	
	≥ 5 mm	64 (5.5)	96.2 (95.3–97.1)	96.3 (95.4–97.1)	98.3 (97.6–99.0)	
CT kernel*	Bone	268 (22.9)	92.1 (91.5–92.7)	92.0 (91.4–92.6)	99.5 (99.4–99.5)	0.623
	Soft tissue	901 (77.1)	92.6 (92.2–92.9)	92.6 (92.3–93.0)	99.3 (99.3–99.4)

**Table 3:** MR image performance metrics by confounding factors. *n* = number of studies (*series). The *p*-value for the median chi-square is provided to determine if a significant difference in accuracy is found for each confounding factor. **124 studies did not have institution information. ***137 series did not have any of the preset sequence tags. Due to the small number of cases, the performance metrics and confidence interval are not reliable for “In and Out of Phase”

	**Category**	***n* (%)**	**F1 (95% CI)**	**Sensitivity (95% CI)**	**Specificity (95% CI)**	***p*-value**
**Institution**	Primary care hospital	435 (54.9)	92.4 (92.0–92.)	92.4 (92.1–92.8)	99.2 (99.1–99.2)	9.7e − 16
	Community hospital	164 (20.7)	90.9 (90.3–91.5)	91.0 (90.4–91.5)	98.5 (98.4–98.7)	
	Imaging center	69 (8.7)	93.8 (92.8–94.8)	93.9 (92.8–94.8)	99.6 (99.6–99.7)	
	Unknown**	124 (15.7)	94.6 (93.6–95.5)	94.4 (93.5–95.4)	99.3 (99.1–99.5)	
**Age**	18–44 years	207 (26.1)	95.7 (95.3–96.2)	95.8 (95.3–96.2)	99.6 (99.6–99.7)	2.1e − 29
	45–64 years	334 (42.2)	91.3 (90.9–91.8)	91.4 (90.9–91.8)	99.2 (99.2–99.3)	
	≥ 65 years	251 (31.7)	90.9 (90.5–91.4)	90.9 (90.5–91.4)	98.7 (98.6–98.8)	
**Sex**	Female	434 (56.1)	92.0 (91.6–92.3)	92.0 (91.6–92.3)	99.1 (99.0–99.1)	0.014
	Male	339 (43.9)	92.5 (92.1–93.0)	92.6 (92.2–93.0)	99.3 (99.2–99.3)	
**Manufacturer**	GE	131 (16.5)	90.3 (89.7–91.0)	90.3 (89.7–91.0)	98.1 (97.9–98.3)	6.4e − 21
	Hitachi	6 (0.8)	NA	NA	NA	
	Philips	40 (5.0)	95.1 (94.3–95.8)	95.1 (94.3–95.8)	98.7 (98.5––99.0)	
	Siemens	565 (71.2)	93.0 (92.7–93.3)	93.0 (92.7–93.3)	99.4 (99.4–99.4)	
	Toshiba	50 (6.3)	93.4 (92.1–94.7)	93.4 (92.1–94.7)	99.6 (99.4–99.7)	
Contrast*	With contrast	343 (16.0)	91.9 (91.3–92.6)	91.9 (91.3–92.6)	98.8 (98.7–98.9)	0.115
	Without contrast	1805 (84.0)	92.3 (92.0–92.5)	92.3 (92.0–92.6)	99.2 (99.2–99.3)	
Slice thickness*	≤ 2 mm	114 (5.3)	98.7 (98.1–99.1)	98.7 (98.2–99.1)	99.9 (99.9–100.0)	2.4e − 27
	> 2 mm and < 5 mm	1472 (68.5)	91.9 (91.5–92.3)	91.9 (91.6–92.3)	99.2 (99.2–99.3)	
	≥ 5 mm	562 (26.2)	91.5 (91.0–92.0)	91.5 (91.0–92.0)	98.7 (98.6–98.8)	
MRI Sequence*	Image weighting	1499 (69.8)	93.1 (92.8–93.5)	93.1 (92.8–93.5)	99.6 (99.6–99.6)	5.0e − 116
	Spin echo	63 (2.9)	92.0 (90.3–93.5)	92.0 (90.3–93.6)	96.2 (95.2–97.2)	
	Gradient echo	246 (11.5)	90.2 (89.6–91.0)	90.3 (89.7–91.0)	97.0 (96.8–97.3)	
	Inversion recovery	116 (5.4)	94.5 (93.3–95.7)	94.5 (93.3–95.7)	99.6 (99.5–99.7)	
	MRA	39 (1.8)	94.9 (92.9–96.7)	94.8 (92.8–96.6)	97.2 (96.0–98.2)	
	In and Out of Phase	14 (0.7)	NA	NA	NA	
	Diffusion	34 (1.6)	82.2 (79.3–83.6)	82.3 (80.2–84.3)	90.1 (88.7–91.4)	
	Unknown***	137 (6.4)	94.2 (93.6–94.8)	94.1 (93.5–94.7)	97.9 (97.6–98.2)	

### Model Performance

An overall body region image-level sensitivity of 92.5% (92.1–92.8) was achieved for CT and 92.3% (92.0–95.6) for MRI. The post-processing stages contributed to about 1.1% (CT) to 1.6% (MRI) improvement in classification accuracy. Classification results by body region and confusion matrices by modality are respectively reported in Table 4 and in Figs. 3 and 4. Head and breast images have very discernible features, so they tend to be classified more accurately than other body regions such as the neck and extremities.

**Table 4:** CT and MR image classification sensitivity and specificity by body region. *n* = number of images

	**CT**				**MRI**			
**Body Region**	***n***	**F1 (95% CI)**	**Sensitivity (95% CI)**	**Specificity (95% CI)**	***n***	**F1 (95% CI)**	**Sensitivity (95% CI)**	**Specificity (95% CI)**
Overall	262,326	92.5 (92.2–92.8)	92.5 (92.1–92.8)	99.4 (99.4–99.5)	118,829	92.2 (91.9–92.5)	92.3 (92.0–92.5)	99.2 (99.1–99.2)
Abdomen	22,302	96.2 (93.1–98.6)	96.7 (96.1–97.3)	98.7 (98.6–98.8)	17,517	90.1 (89.6–90.6)	92.4 (91.7–93.0)	97.5 (97.3–97.6)
Arm	14,430	93.9 (89.1–97.5)	94.1 (92.9–95.3)	99.2 (99.1–99.3)	3815	91.6 (90.5–92.7)	88.8 (87.1–90.5)	99.8 (99.8–99.8)
Breast	-	-	-	-	20,501	100.0 (100.0–100.0)	100.0 (100.0–100.0)	100.0 (100.0–100.0)
Calf	12,308	94.7 (91.9–97.0)	93.0 (91.7–94.3)	99.7 (99.6–99.7)	3299	93.2 (92.2–94.3)	95.7 (94.5–96.9)	99.6 (99.6–99.7)
Chest	27,968	96.4 (93.5–98.7)	96.9 (96.3–97.6)	98.9 (98.8–99.0)	13,010	89.6 (88.9–90.2)	89.2 (88.4–90.1)	98.5 (98.4–98.7)
Cervical spine	11,993	87.1 (85.4–88.8)	78.0 (76.0–80.0)	99.9 (99.9–99.9)	3607	75.0 (72.6–77.5)	62.3 (59.3–65.5)	99.9 (99.9–99.9)
Elbow	11,288	91.6 (87.5–94.6)	88.0 (86.0–90.0)	99.6 (99.6–99.7)	4615	91.8 (90.7–92.9)	91.2 (89.6–92.7)	99.7 (99.6–99.7)
Foot	13,137	92.0 (90.2–93.6)	86.5 (84.8–88.2)	99.9 (99.8–99.9)	3178	95.3 (94.2–96.4)	94.8 (93.3–96.4)	99.9 (99.9–99.9)
Forearm	8232	89.3 (83.9–93.8)	86.6 (84.3–88.8)	99.5 (99.5–99.6)	3546	88.8 (87.4–90.1)	86.0 (84.1–87.9)	99.7 (99.7–99.8)
Hand	11,321	95.0 (91.4–97.9)	94.9 (93.7–96.2)	99.6 (99.5–99.6)	6699	95.9 (95.2–96.6)	96.3 (95.4–97.3)	99.8 (99.7–99.8)
Head	29,066	99.1 (98.7–99.5)	98.7 (98.3–99.1)	99.9 (99.9–99.9)	7633	97.3 (96.7–97.9)	99.3 (98.8–99.7)	99.7 (99.7–99.8)
Knee	10,721	93.7 (89.0–97.5)	94.0 (92.7–95.3)	99.4 (99.3–99.4)	3649	95.2 (94.3–96.2)	95.8 (94.5–97.0)	99.8 (99.8–99.9)
Lumbar spine	16,100	95.9 (94.4–97.2)	93.6 (92.4–94.8)	99.8 (99.8–99.9)	4556	96.5 (95.7–97.2)	97.0 (96.0–98.0)	98.8 (99.8–99.9)
Neck	19,292	93.4 (88.1–97.6)	94.4 (93.3–95.5)	98.9 (98.8–99.0)	4363	77.5 (75.5–79.3)	86.1 (83.7–88.6)	98.9 (98.8–99.0)
Pelvis	13,886	90.9 (89.0–92.6)	84.9 (83.2–86.6)	99.8 (99.7–99.8)	11,632	92.3 (91.6–92.9)	90.3 (89.4–91.3)	99.4 (99.3–99.5)
Shoulder	13,266	92.7 (90.3–94.7)	88.6 (86.9–90.2)	99.8 (99.7–99.8)	5599	95.4 (94.7–96.2)	98.6 (98.0–99.2)	99.6 (99.6–99.7)
Thigh	14,409	94.0 (92.8–95.1)	89.4 (88.0–90.8)	99.9 (99.9–99.9)	4485	93.4 (92.4–94.3)	88.9 (87.3–90.5)	99.9 (99.9–100.0)
Thoracic spine	21,378	94.4 (92.5–96.0)	91.4 (90.3–92.5)	99.5 (99.5–99.6)	3952	93.0 (91.9–94.1)	93.4 (91.9–94.8)	99.7 (99.7–99.8)

No formal association was found between classification accuracy and CT institution, CT kernel, and MRI contrast. However, statistically superior classification results were noticed in a few instances with Cramer’s *V* correlation ranging from negligible (*V* < 0.05) to moderate (*V* = 0.17). For CT, that was the case for datasets with older (≥ 65) age (*p* < 0.001, *V* = 0.041) with contrast (*p* < 0.001, *V* = 0.042) and thick (≥ 5 mm) slice (*p* =  < 0.001, *V* = 0.048). For MRI, imaging centers (*p* < 0.001, *V* = 0.064), 44 years and older (*p* < 0.001, *V* = 0.087), Philips manufacturer (*p* = 0.001, *V* = 0.076), thin slices (*p* < 0.001, *V* = 0.0838), and inversion and MRA sequences (*p* < 0.001, *V* = 0.179) exhibited better classification performance. For some of the classes in the test sets, the association between accuracy and factors such as manufacturer and MRI sequence could not be reliably assessed: Hitachi and Canon scanner manufacturers and In and Out of Phase MRI sequences. Despite these limitations, the evaluation of accuracy results and confidence intervals points to performance robustness across age, manufacturer, CT slice thickness, and MRI sequence categories.

When mining the DICOM tags in the test datasets for either the “BodyPartExamined” DICOM tag (BP) or “ProcedureType” (PT), the body region information at the study level was only 22.3% (BP) and 42.2% (PT) accurate for CT and 58.3% (BP) and 47.8% (PT) accurate for MRI. In this cohort, the anatomical AI could prove useful to improve the search for anatomically matched cases for about 50% of the cases.

## Discussion

The ability to automate accurate anatomic region labeling of medical images using pixel-based AI could address clinical and research workflow challenges related to existing limitations that affect body region labeling of medical images. Our work demonstrates how a deep learning CNN-based classifier can achieve overall state-of-the-art accuracy greater than 90% in identifying body regions in CT and MR images while covering the entire human body and a large spectrum of acquisition protocols obtained from separate institutions. This is the first known attempt to (a) provide a solution that includes MR and (b) offers a solution that covers numerous body regions, in particular extremities that have been excluded in other CT studies.

Our methods achieved an overall body region image-level sensitivity of 92.5% which were similar to other publications restricted to CT image classification [2–6]. In contrast to all other studies, this algorithm can label both CT and MR images and therefore provides a potential solution for improved study labeling to support complex image interpretation workflows that demand evaluation of equivalent body regions across several cross-sectional imaging modalities, including whole-body MR, PET CT, and PET MR. Additionally, generalizability of this technique across modality vendors and multiple different imaging protocols is also considered relevant to address the increasing need for standardized anatomic labeling of multi-institutional datasets.

Our results demonstrate that confidence intervals for sensitivity were lower (upper bound did not include 90%) for the following specific anatomical regions: CT cervical spine, CT forearm, CT pelvis, CT foot, MRI cervical spine, MRI forearm, and MRI neck. Concentrations of model misclassifications were observed in the transitions between body regions, for example: abdomen label assigned to source of truth labeled “pelvis” or chest label assigned to source of truth labeled “cervical spine.” The high prevalence of these images, related to scan acquisition techniques, contributes to the lower model performance and wider confidence interval. Examples of erroneous predictions in transition areas can be found in the Supplemental Materials Examples of ML Misclassifications (Figs. 5–7). Additionally for neck and cervical spine, the visual differentiation between a neck and a cervical spine study on soft tissue reconstructions is sometimes minimal resulting in ground truth labeling inconsistencies. This may have contributed to lower model performance for these body regions.

We observed several challenges that affected accuracy of body region classification in extremities. First, extremities are sometimes imaged in orientations that are influenced by the patient’s oblique positioning in the CT gantry or unconventional scan angle protocols for MR. Although the training dataset was developed to account for a diversity of extremity orientations, unconventional orientations may have had an adverse effect on model performance. Second, extremities are subject to fractures, surgical implants, and amputation, which can result in deformities that could potentially cause body region misclassification. Third, images of upper and lower extremities have structural similarities that resulted in misclassification of some images of paired long bones of the lower leg and forearm, single long bones of the thigh and arm, and knee and elbow. Fourth, the landmarks’ positions between the defined body regions for the upper and lower extremities are more subjective when bones are not fully present in the images (example: proximal 6th of the humerus) than between other body regions resulting in some model misclassifications in the transition zones.

Our study has some limitations. First, the multi-class framework is, by design, not well suited to identify multiple regions in an image. This is a limitation when dealing with whole-body studies (the shoulders, arms, elbows, forearms, and hands are sometimes included in chest, abdomen, and pelvis images) and images that often include two body regions, for example, shoulders included in chest images. Second, there is under-representation of several classes in the datasets, for example, Hitachi and Canon scanner manufacturers and In and Out of Phase MRI sequences. Additional data and analysis is needed to complete the evaluation for these classes.

As stated in a joint paper by HIMSS and SIIM, the implications of our work are multiple [1]. An improved method for anatomic labeling of imaging studies has the potential to improve interoperability across healthcare records and systems, address radiology workflow challenges such as labeling discrepancies for studies shared within and between facilities [17], improve accurate retrieval of anatomically relevant comparison images from image archives, and potentially reduce bandwidth-related latency and costs associated with unnecessary data retrieval from cloud-based image archives. Application of our body region labeling has the potential to improve display protocols, image synchronization, and relevant prior functions in PACS, leading to improvements in image-based diagnosis and treatments, especially in complex patients. When applied to increasingly large volumes of radiology exams per year (millions), even small improvements in image based workflows have the potential for large and safety quality impacts. Beyond image interpretation, this technique could enable body region-dependent AI-driven population health initiatives across institutions.

Further research is suggested to investigate the effects of merging overlapping classes, for example, the neck and cervical spine, and consider exclusion methods to address labeling issues related to transitional anatomy and image obliquity. Other opportunities include development of a multi-label classification approach and extending the application and evaluation of this technique to other imaging data sources. Additionally, observational studies are required to assess the clinical value of this technique, specifically for complex clinical image interpretation workflows.

Reliable selection and presentation of comparison of images matched by body parts is an essential function of image interpretation systems. Accurate and standardized body region labeling challenges, resulting from consolidation of services and development of large multi-institutional imaging datasets, can adversely affect accurate image-based diagnosis and management of complex patients.

Automatic identification of body regions in CT and MR studies in a general population is a challenging task due to the diverse spectrum of demographics, comorbidities, acquisition protocols, and imaging artifacts. Our research demonstrates that our anatomical AI technique can provide state-of-the-art image-level classification for CT and MR with an accuracy greater than 90% and performance metrics robust across all body regions and confounding factors such as institution, sex, contrast, manufacturer, slice thickness, CT kernel, and MRI sequences.

## Supplementary Information

Below is the link to the electronic supplementary material.

Supplementary file1 (DOCX 1449 KB)
