# Supplementary material for: Deep Learning Body Region Classification of MRI and CT Examinations
Source: J Digit Imaging. 2023 Mar 9;36(4):1291–301. doi: 10.1007/s10278-022-00767-9 (PMC10407003; doi:10.1007/s10278-022-00767-9)
Supplement: Supplementary file 1 — Supplementary file1 (DOCX 1449 KB) [file 10278_2022_767_MOESM1_ESM.docx]

SUPPLEMENTAL MATERIALS

**Deep Learning Body Region Classification of MRI and CT examinations**

[Inclusion and Exclusion Criteria 2](#_Toc112661471)

[MRI Sequences 2](#_Toc112661472)

[Flowchart of Exclusion Criteria (CT Development Datasets) 4](#_Toc112661473)

[Flowchart of Exclusion Criteria (MRI Development Datasets) 5](#_Toc112661474)

[Flowchart of Exclusion Criteria (CT Test Dataset) 6](#_Toc112661475)

[Flowchart of Exclusion Criteria (MRI Test Dataset) 7](#_Toc112661476)

[Distribution of Development Datasets 8](#_Toc112661477)

[Patient and Image Distribution by Body Region (CT) 8](#_Toc112661478)

[Patient and Image Distribution by Body Region (MRI) 9](#_Toc112661479)

[Study Distribution by Demographics, and Manufacturer (CT) 10](#_Toc112661480)

[Series Distribution by Scanner Parameters and Clinical Protocol (CT) 11](#_Toc112661481)

[Study Distribution by Demographics and Manufacturer (MRI) 12](#_Toc112661482)

[Series Distribution by Scanner Parameters and Clinical Protocol (MRI) 13](#_Toc112661483)

[Results Validation Dataset 14](#_Toc112661484)

[Evaluation Metrics 14](#_Toc112661485)

[Sensitivity and Specificity by Body Region (CT) 15](#_Toc112661486)

[Sensitivity and Specificity by Body Region (MRI) 16](#_Toc112661487)

[Sensitivity and Specificity by Demographics, Manufacturer, and Scanner parameters (CT) 17](#_Toc112661488)

[Sensitivity and Specificity by Demographics, Manufacturer, and Scanner parameters (MRI) 18](#_Toc112661489)

[Examples of ML Misclassifications 19](#_Toc112661490)

[Misclassifications in Anatomical Transition Areas 19](#_Toc112661491)

[Other Image Misclassifications 20](#_Toc112661492)

[Training Parameters 21](#_Toc112661493)

[Performance and Hardware Information 22](#_Toc112661494)

# Inclusion and Exclusion Criteria

## MRI Sequences

Many types of MRI sequences were included in the study such as image weighting, spin echo, gradient echo, inversion recovery**, MRA, In and Out of Phase, and diffusion imaging (Table 1).**

**Were excluded from consideration in the study, imaging sequences used to determine flow velocities** such as VIPR (High Speed 3D Phase-Contrast method for flow) and FASTPC (Fast Phase Contrast Imaging) as well as the dynamic MRA sequence called TRICKS (Time Resolved Imaging of Contrast KineticS) and MRE (MR Elastography) used for liver disease. Regarding CT, only series related to the evaluation of flow velocities were excluded. For both modalities, a first filtering pass was applied using a search on selective keywords in the series description (Table 2). A second quality control review was conducted to ensure no irrelevant series were kept in the training dataset.

| **MRI Sequence Type** | **Sequence Names** |
| --- | --- |
| Image weighting | T1, T2, T1/T2*, PD, SWI, SWAN, BRAVO, MERGE |
| Spin echo | SE, Fast SE, Single Shot FSE, HASTE, VISTA, PROPELLER |
| Gradient echo | GRE, FFE, FE, SPGR, FGRE, LAVA, VIBRANT, VIBE, BLISS, FISP, SSFP, FIESTA, TRU FISP |
| Inversion recovery | IR, STIR, FLAIR |
| MRA | TOF, CONTRAST ENHANCED, DELAY ENHANCED |
| In and Out of Phase | IN/OUT, IN of Phase, Out of Phase |
| Diffusion | DIFFUSION, DWI |

**Table 1**: Examples of MRI sequences included in the development datasets.

|  | **EXCLUDED SEQUENCES WITH FOLLOWING KEYWORDS** |
| --- | --- |
| **MRI series** | FLOW, VELOCITY, ADC, APPARENT DIFFUSION, IDEAL, EP2D_DIFF, FASTPC, PC, CBV, CBF, MTT, TTP, CAD, DWI_SSH, VIPR |
| **CT series** | Velocity |

**Table 2**: Series filtering using selective keywords in the series description.

## Flowchart of Exclusion Criteria (CT Development Datasets)


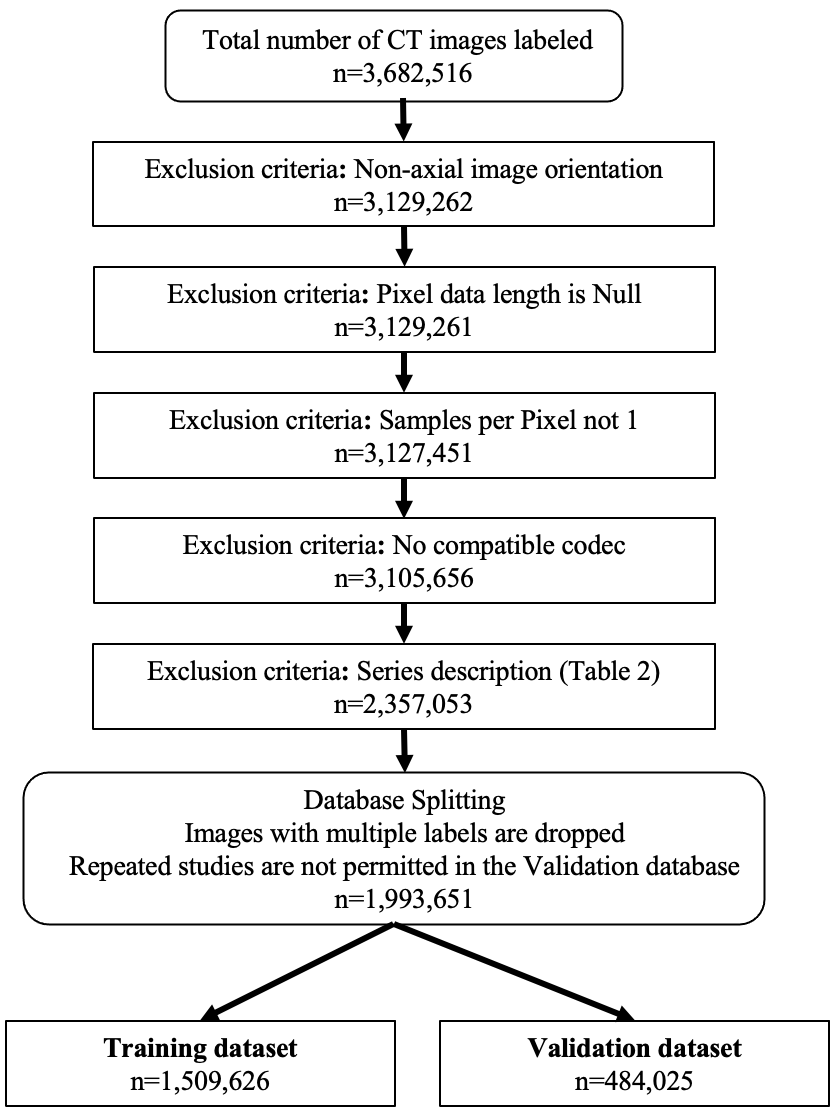


**Figure 1**: Flowchart of exclusion criteria for CT annotated images (development datasets).

## Flowchart of Exclusion Criteria (MRI Development Datasets)


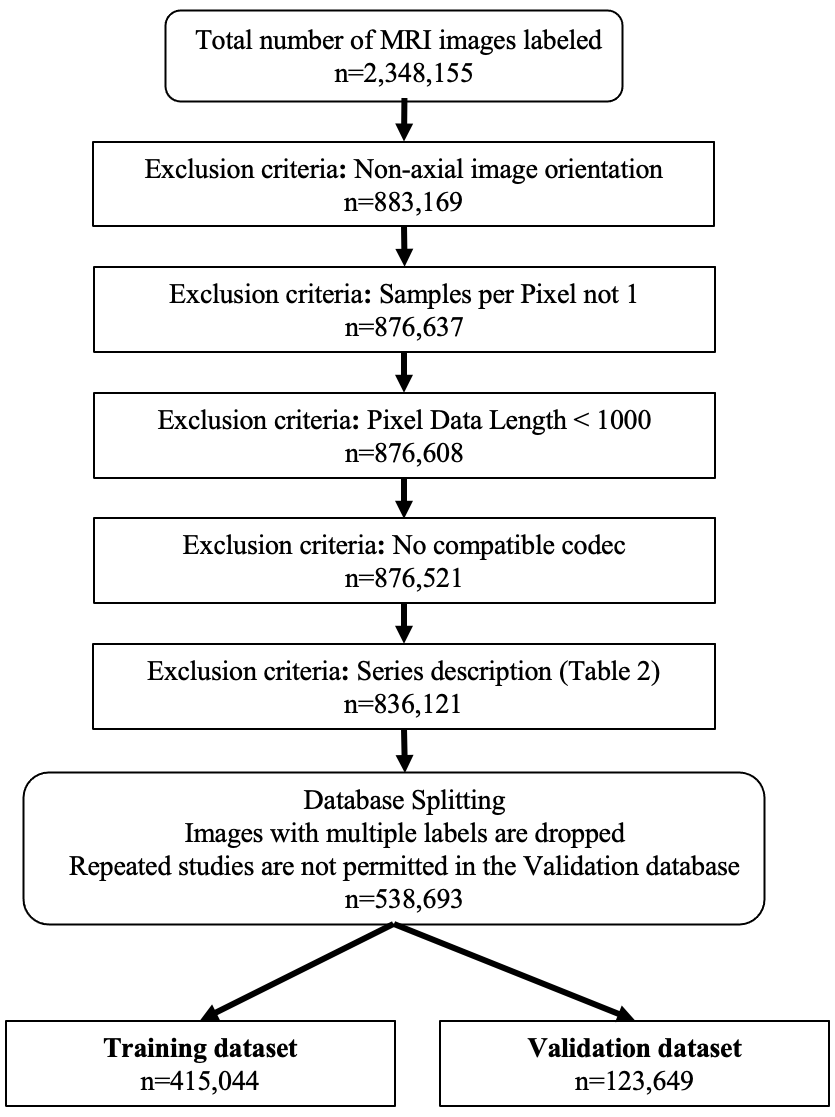


**Figure 2**: Flowchart of exclusion criteria for MRI annotated images (development datasets).

## Flowchart of Exclusion Criteria (CT Test Dataset)


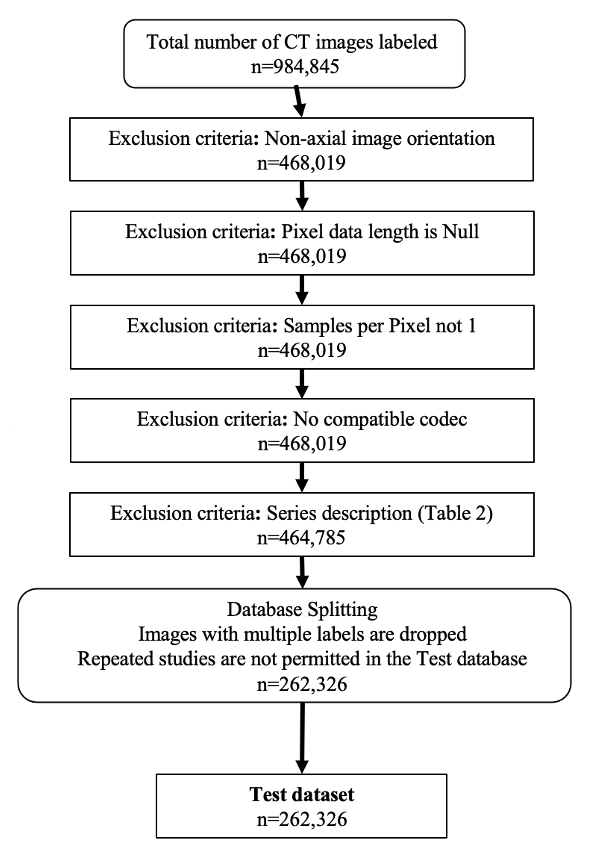


**Figure 3**: Flowchart of exclusion criteria for CT annotated images (Test dataset).

## Flowchart of Exclusion Criteria (MRI Test Dataset)


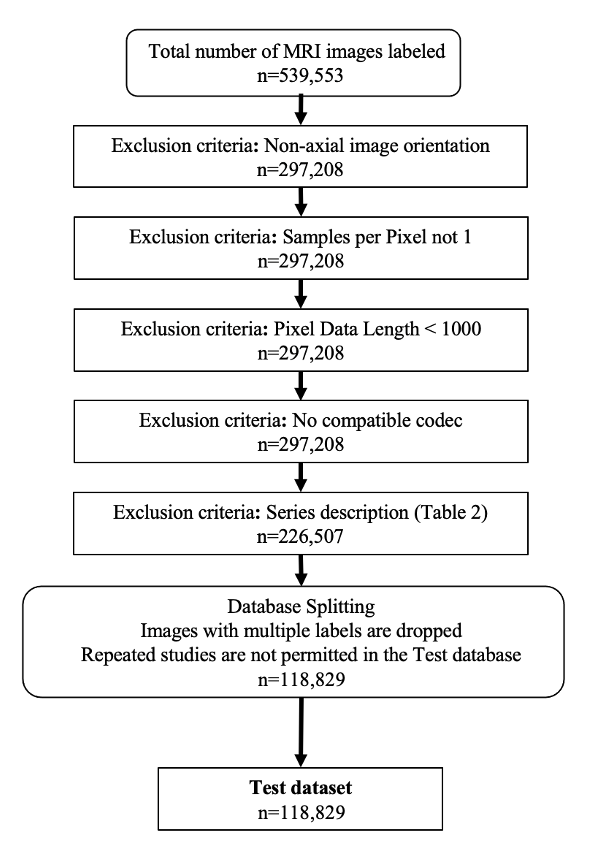


**Figure 4**: Flowchart of exclusion criteria for MRI test annotated images (Test dataset).

# Distribution of Development Datasets

## Patient and Image Distribution by Body Region (CT)

| **Body Region** | **Patients** | | **Images** | |
| --- | --- | --- | --- | --- |
|  | Training | Validation | Training | Validation |
| Head | 262 | 86 | 78,487 | 20,732 |
| Neck | 315 | 107 | 54,691 | 17,483 |
| Cervical spine | 209 | 68 | 52,470 | 17,467 |
| Chest | 510 | 165 | 105,262 | 34,745 |
| Thoracic spine | 285 | 95 | 74,552 | 24,566 |
| Abdmchst | 356 | 117 | 48,660 | 17,854 |
| Abdomen | 354 | 113 | 58,808 | 19,154 |
| Lumbar spine | 147 | 49 | 65,147 | 21,098 |
| Pelvis | 319 | 96 | 62,556 | 19,018 |
| Thigh | 228 | 69 | 88,390 | 25,663 |
| Knee | 227 | 70 | 131,590 | 43,054 |
| Calf | 262 | 82 | 150,307 | 46,935 |
| Foot | 245 | 81 | 149,354 | 47,647 |
| Shoulder | 290 | 102 | 70,008 | 24,832 |
| Arm | 361 | 126 | 72,357 | 23,209 |
| Elbow | 237 | 82 | 77,044 | 25,847 |
| Forearm | 254 | 90 | 49,548 | 17,515 |
| Hand | 250 | 79 | 120,395 | 37,206 |

**Table 4**: Distribution of CT patients as a function of body region information extracted from the ground-truth. Note that a study can contain several body regions, as a result it may contribute to several anatomical regions.

## Patient and Image Distribution by Body Region (MRI)

| **Body Region** | **Patients** | | **Images** | | |
| --- | --- | --- | --- | --- | --- |
|  | Training | Validation | Training | Validation | |
| Head | 208 | 66 | 60,027 | 17,474 | |
| Neck | 249 | 84 | 15,838 | 4,923 | |
| Cervical spine | 135 | 43 | 5,976 | 1,989 | |
| Chest | 330 | 108 | 17,995 | 4,895 | |
| Thoracic spine | 185 | 63 | 98,27 | | 3,226 |
| Abdmchst | 265 | 89 | 18,672 | | 5,649 |
| Abdomen | 253 | 78 | 40,835 | | 11,680 |
| Lumbar spine | 134 | 45 | 7,660 | | 2,094 |
| Pelvis | 288 | 96 | 31,777 | | 8,783 |
| Thigh | 201 | 55 | 11,795 | | 3,272 |
| Knee | 219 | 74 | 7,898 | | 2,425 |
| Calf | 138 | 44 | 11,160 | | 3,701 |
| Foot | 224 | 75 | 10,433 | | 3,082 |
| Shoulder | 176 | 62 | 6,380 | | 1,945 |
| Arm | 180 | 61 | 7,844 | | 2,432 |
| Elbow | 325 | 100 | 16,926 | | 4,911 |
| Forearm | 225 | 72 | 11,342 | | 3,778 |
| Hand | 223 | 69 | 14,263 | | 4,234 |
| Breast | 137 | 46 | 108,396 | | 33,156 |

**Table 5**: Distribution of MRI patients as a function of body region information extracted from the ground-truth. Note that a study can contain several body regions, as a result it may contribute to several anatomical regions.

## Study Distribution by Demographics, and Manufacturer (CT)

| **Attributes** | **Categories** | **Training** | | **Validation** | |
| --- | --- | --- | --- | --- | --- |
|  |  | n | % | n | % |
| **Sex** | Female | 867 | 48.1 | 294 | 48.8 |
|  | Male | 935 | 51.9 | 308 | 51.2 |
| **Age** | 18 - 44 years | 436 | 24.2 | 133 | 22.1 |
|  | 45 – 64 years | 640 | 35.5 | 236 | 39.3 |
|  | >= 65 years | 654 | 36.3 | 212 | 35.3 |
| **Manufacturer** | Canon | - | - | - | - |
|  | GE | 1,671 | 92.5 | 555 | 92.3 |
|  | Hitachi | - | - | - | - |
|  | Philips | 4 | 0.2 | 2 | 0.3 |
|  | Siemens | 96 | 5.3 | 37 | 6.2 |
|  | Toshiba | 35 | 1.9 | 7 | 1.2 |
|  | Others | - | - | - | - |

**Table 6**: Distribution of CT studies with respect to demographics, and manufacturer.

## Series Distribution by Scanner Parameters and Clinical Protocol (CT)

| **Attributes** | **Categories** | **Training** | | **Validation** | |
| --- | --- | --- | --- | --- | --- |
|  |  | n | % | n | % |
| **Contrast** | With contrast | 1,300 | 32.6 | 404 | 31.1 |
|  | Without contrast | 2689 | 67.4 | 894 | 68.9 |
| **Slice thickness** | <= 2 mm | 2,722 | 67.2 | 899 | 68.1 |
|  | >2 mm and < 5 mm | 343 | 8.5 | 98 | 7.4 |
|  | >= 5 mm | 985 | 24.3 | 324 | 24.5 |
| **Kernel** | Bone | 1,072 | 26.9 | 342 | 26.3 |
|  | Soft tissue | 2,917 | 73.1 | 956 | 73.7 |

**Table 7**: Distribution of CT series with respect to clinical protocol. Series with a bone CT kernel were identified using a search of the keyword “bone” in the series description. It includes series with and without Metal Artifact Reduction (MAR). All other axial series in the same study were considered soft tissue acquisitions. The studies with contrast include a spectrum of contrast phases, such as portal venous phase, arterial phase, and delayed phase imaging.

## Study Distribution by Demographics and Manufacturer (MRI)

| **Attributes** | **Categories** | **Training** | | | **Validation** | |
| --- | --- | --- | --- | --- | --- | --- |
|  |  | n | % | n | | % |
| **Sex** | Female | 1,040 | 54.5 | 326 | | 52.2 |
|  | Male | 870 | 45.5 | 298 | | 47.8 |
| **Age** | 18 – 44 years | 592 | 31.6 | 194 | | 31.5 |
|  | 45 – 64 years | 668 | 35.6 | 214 | | 34.3 |
|  | >= 65 years | 375 | 20 | 130 | | 21.1 |
| **Manufacturer** | GE | 1,634 | 85.5 | 542 | | 86.7 |
|  | Hitachi | - | - | 2 | | 0.3 |
|  | Philips | 40 | 2.1 | 12 | | 1.9 |
|  | Siemens | 235 | 12.3 | 69 | | 11 |
|  | Toshiba | 1 | 0.1 | - | | - |

**Table 8**: Distribution of MRI studies with respect to demographics, and manufacturer.

## Series Distribution by Scanner Parameters and Clinical Protocol (MRI)

| **Attributes** | **Categories** | **Training** | | **Validation** | |
| --- | --- | --- | --- | --- | --- |
|  |  | n | % | n | % |
| **Contrast** | With contrast | 1585 | 22.4 | 520 | 24.1 |
|  | Without contrast | 5466 | 77.6 | 1641 | 75.9 |
| **Slice thickness** | <= 2 mm | 934 | 13.2 | 287 | 13.3 |
|  | >2 mm and < 5 mm | 3,402 | 48.2 | 1,102 | 51.0 |
|  | >= 5 mm | 2,715 | 38.5 | 772 | 35.7 |
| **Sequences** | Image weighting | 5,378 | 73.2 | 1,656 | 73.9 |
|  | Spin echo | 329 | 4.5 | 127 | 5.7 |
|  | Gradient echo | 821 | 11.2 | 234 | 10.4 |
|  | Inversion recovery | 221 | 3.0 | 57 | 2.5 |
|  | MRA | 182 | 2.5 | 40 | 1.8 |
|  | In and Out of Phase | 108 | 1.5 | 38 | 1.7 |
|  | Diffusion | 307 | 4.2 | 88 | 3.9 |

**Table 9**: Distribution of MRI series with respect to clinical protocol. MRI sequences are selected based on the search of keywords in series description listed in Table 1.

# Results Validation Dataset

## Evaluation Metrics

Sensitivity and specificity were used as the primary evaluation metrics as required by the FDA for the 510K submission. These metrics measure the true positive rate, sensitivity, and the true negative rate. This allows the end user to have direct knowledge of how likely a positive or negative prediction is to be correct. It is also the standard required by the FDA 510K submission process for such models. The F1 score is a balanced accuracy score attempting to summarize the precision and sensitivity into one number. The reported aggregate metrics evenly weight all classes.

Overall accuracy was not reported as it is strongly influenced by the number of images from each class. Since some body regions are more frequently imaged than others, using the accuracy would yield results relevant for the main classes but fail to show how the less frequent classes perform. An average weighted by the number of images of the class level sensitivity is the same as the overall accuracy. The class balance of the test set may not represent all uses cases, as such class level metrics are preferred for comparison and evaluation.

$$Sensitivity=\frac{TruePositives}{TruePositive+FalseNegitives}$$

$$Specificity=\frac{TrueNegatives}{TrueNegatives+FalseNegitives}$$

$$F_{1}Score=\frac{2*TruePostives}{2*TruePostives+FalsePostives+FalseNegatives}$$

## Sensitivity and Specificity by Body Region (CT)

| **Body Region** | **Sensitivity (95% CI)** | **Specificity (95% CI)** |
| --- | --- | --- |
| Overall | 91.7 (91.5 – 91.9) | 99.3 (99.3 – 99.4) |
| Head | 98.7 (98.3 – 99.1) | 99.9 (99.8 – 99.9) |
| Neck | 89.4 (88.1 – 90.7) | 99.8 (99.7 – 99.8) |
| Cervical spine | 91.6 (90.1 – 93.1) | 99.8 (99.7 – 99.8) |
| Chest | 92.6 (92.0 – 93.1) | 98.9 (98.8 – 98.9) |
| Thoracic spine | 95.6 (94.7 – 96.6) | 99.6 (99.5 – 99.6) |
| Abdomen | 93.3 (92.8 – 93.9) | 98.8 (98.7 – 98.9) |
| Lumbar spine | 96.4 (95.5 – 97.2) | 99.8 (99.8 – 99.9) |
| Pelvis | 90.5 (89.4 – 91.5) | 99.5 (99.5 – 99.6) |
| Thigh | 88.2 (86.9 – 89.4) | 99.3 (99.2 – 99.4) |
| Knee | 82.1 (80.7 – 83.4) | 99.5 (99.4 – 99.5) |
| Calf | 91.2 (90.2 – 92.2) | 99.3 (99.2 – 99.3) |
| Foot | 94.2 (93.4 – 95.1) | 99.6 (99.6 – 99.7) |
| Shoulder | 91.4 (90.3 – 92.3) | 99.5 (99.4 – 99.5) |
| Arm | 90.4 (89.2 – 91.5) | 99.1 (99.0 – 99.2) |
| Elbow | 85.6 (84.1 – 87.2) | 99.6 (99.5 – 99.6) |
| Forearm | 87.2 (85.6 – 88.8) | 99.4 (99.3 – 99.5) |
| Hand | 92.4 (91.5 – 93.4) | 99.8 (99.8 – 99.8) |

**Table 10**: Validation dataset - CT weighted image-level sensitivity and specificity (%) as a function of body region.

## Sensitivity and Specificity by Body Region (MRI)

| **Body Region** | **Sensitivity (95% CI)** | **Specificity (95% CI)** |
| --- | --- | --- |
| Overall | 93.9 (93.7 – 94.3) | 99.3 (99.3 – 99.3) |
| Head | 98.9 (98.4 – 99.4) | 99.6 (99.5 – 99.7) |
| Neck | 85.4 (82.7 – 88.0) | 99.8 (99.8 – 99.9) |
| Cervical spine | 84.8 (81.9 – 87.7) | 99.9 (99.9 – 99.9) |
| Chest | 89.6 (88.7 – 90.6) | 98.5 (98.3 – 98.6) |
| Thoracic spine | 97.9 (97.0 – 98.8) | 99.7 (99.6 – 99.8) |
| Abdomen | 92.7 (92.2 – 93.6) | 98.0 (97.9 – 98.2) |
| Lumbar spine | 89.8 (87.3 – 92.0) | 99.8 (99.8 – 99.9) |
| Pelvis | 92.1 (91.0 – 93.2) | 99.7 (99.6 – 99.7) |
| Thigh | 91.7 (90.0 – 93.4) | 99.9 (99.9 – 99.9) |
| Knee | 94.4 (92.6 – 96.0) | 99.7 (99.7 – 99.8) |
| Calf | 90.8 (89.1 – 92.7) | 99.8 (99.7 – 99.8) |
| Foot | 94.9 (93.2 – 96.6) | 99.8 (99.8 – 99.9) |
| Shoulder | 97.1 (95.6 – 98.5) | 99.9 (99.9 – 99.9) |
| Arm | 90.2 (87.9 – 92.3) | 99.8 (99.7 – 99.8) |
| Elbow | 92.3 (90.5 – 94.0) | 99.7 (99.6 – 99.8) |
| Forearm | 91.8 (89.8 – 93.8) | 99.8 (99.8 – 99.9) |
| Hand | 97.5 (96.3 – 98.5) | 99.8 (99.8 – 99.9) |
| Breast | 99.9 (99.8 – 99.9) | 99.9 (99.9 – 99.9) |

**Table 11**: Validation dataset - MRI weighted image-level sensitivity and specificity (%) as a function of body region.

## Sensitivity and Specificity by Demographics, Manufacturer, and Scanner parameters (CT)

| **Attributes** | **Categories** | **Sensitivity (95% CI)** | **Specificity (95% CI)** |
| --- | --- | --- | --- |
| **Sex** | Female | 91.8 (91.4 – 92.1) | 99.4 (99.3 – 99.4) |
|  | Male | 91.7 (91.4 – 92.0) | 99.3 (99.3 – 99.3) |
| **Age** | 18 – 44 years | 90.8 (90.3 – 91.3) | 99.3 (99.3 – 99.4) |
|  | 45 – 64 years | 92.2 (91.9 – 92.5) | 99.3 (99.2 – 99.3) |
|  | >= 65 years | 91.6 (91.1 – 91.9) | 99.4 (99.3 – 99.4) |
| **Manufacturer** | GE | 91.9 (91.7 – 92.2) | 99.3 (99.3 – 99.4) |
|  | Philips | - | - |
|  | Siemens | 89.4 (88.1 – 90.7) | 98.8 (98.6 – 98.9) |
|  | Toshiba | 78.9 (75.3 – 82.4) | 97.9 (97.1 – 98.7) |
| **Contrast** | With contrast | 92.9 (92.6 – 93.3) | 99.0 (98.9 – 99.1) |
|  | Without contrast | 90.9 (90.7 – 91.3) | 99.4 (99.3 – 99.4) |
| **Kernel** | Bone | 91.3 (90.8 – 91.7) | 99.4 (99.4 – 99.4) |
|  | Soft tissue | 91.9 (91.6 – 92.1) | 99.1 (99.1 – 99.2) |
| **Slice thickness** | <= 2 mm | 91.3 (90.9 – 91.5) | 99.4 (99.4 – 99.4) |
|  | > 2 mm and < 5 mm | 91.7 (91.0 – 92.4) | 99.2 (99.1 – 99.3) |
|  | >= 5 mm | 93.4 (92.9 – 93.8) | 96.9 (96.6 – 97.2 |

**Table 12**: Validation dataset - CT weighted image-level sensitivity and specificity (%) as a function of demographics, manufacturer and acquisition protocol.

## Sensitivity and Specificity by Demographics, Manufacturer, and Scanner parameters (MRI)

| **Attributes** | **Categories** | **Sensitivity (95% CI)** | **Specificity (95% CI)** |
| --- | --- | --- | --- |
| **Sex** | Female | 94.9 (94.5 – 95.2) | 99.4 (99.4 – 99.5) |
|  | Male | 92.3 (92.4 – 93.3) | 99.0 (98.9 – 99.1) |
| **Age** | 18 – 44 years | 94.9 (94.4 – 95.4) | 99.5 (99.5 – 99.6) |
|  | 45 – 64 years | 93.9 (93.5 – 94.4) | 99.3 (99.3 – 99.4) |
|  | >= 65 years | 93.0 (92.5 – 93.5) | 98.9 (98.8 – 99.0) |
| **Manufacturer** | GE | 93.9 (93.6 – 94.2) | 99.4 (99.4 – 99.4) |
|  | Hitachi | - | - |
|  | Philips | - | - |
|  | Siemens | 94.1 (93.4 – 94.7) | 98.3 (98.0 – 98.5) |
|  | Toshiba | - | - |
| **Contrast** | With contrast | 94.8 (94.4 – 95.3) | 99.2 (99.1 – 99.3) |
|  | Without contrast | 93.5 (93.2 – 93.9) | 99.3 (99.3 – 99.4) |
| **Slice thickness** | <= 2 mm | 98.4 (97.9 – 98.8) | 98.9 (98.4 – 99.3) |
|  | > 2 mm and < 5 mm | 94.3 (93.6 – 94.5) | 99.5 (99.4 – 99.5) |
|  | >= 5 mm | 92.5 (92.1 – 92.9) | 98.8 (98.6 – 98.8) |
| **Sequences** | Image weighting | 93.5 (93.1 – 93.9) | 99.5 (99.4 – 99.5) |
|  | Spin echo | 91.5 (89.8 – 93.2) | 99.1 (98.9 – 99.3) |
|  | Gradient echo | 95.1 (94.5 – 95.6) | 98.6 (98.5 – 98.8) |
|  | Inversion recovery | 89.6 (86.9 – 92.3) | 99.3 (99.1 – 99.5) |
|  | MRA | 95.9 (93.7 – 97.8) | 97.6 (95.7 – 99.1) |
|  | In and Out of Phase | 91.1 (89.8 – 92.4) | 88.9 (87.1 – 90.9) |
|  | Diffusion | 95.3 (94.3 – 96.3) | 99.0 (98.7 – 99.3) |

**Table 13**: Validation dataset - MRI weighted image-level sensitivity and specificity (%) as a function of demographics, manufacturer, and acquisition protocol.

# Examples of ML Misclassifications

## Misclassifications in Anatomical Transition Areas

Definitions: GT = Ground Truth, MP = Model Prediction.

| 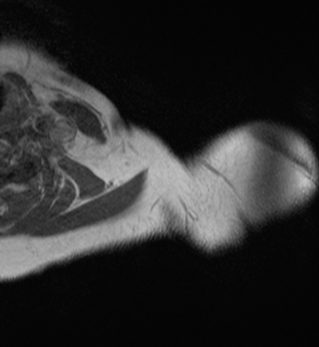  a: GT = neck, MP = neck | 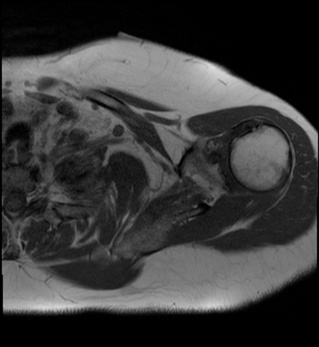  b: GT = neck, MP = shoulder | 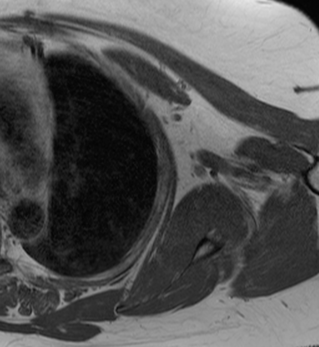  c: GT = chest, MP = chest |
| --- | --- | --- |

**Figure 5**: MRI Chest study. Images correctly inferred as neck (a) and chest (c). The image in-between is classified as shoulder instead of neck (b). The shoulder is clearly present in the image. This illustrates one limitation of the multi-class framework where only one label is assigned to an image.

| 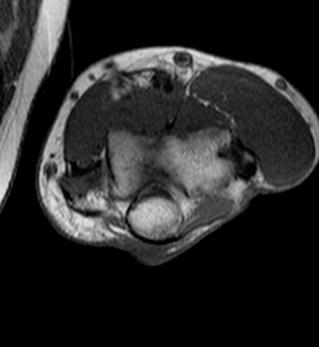  a: GT = elbow, MP = elbow | 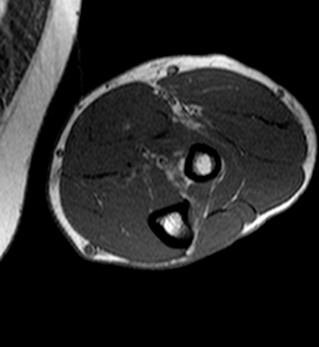  b: GT = forearm, MP = elbow | 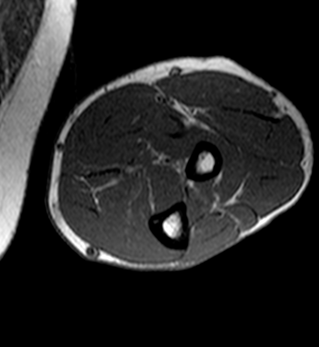  c: GT = forearm, MP = forearm |
| --- | --- | --- |

**Figure 6**: MRI Upper extremity study. Images correctly inferred as elbow (a) and forearm (c). The image in-between is misclassified as elbow (b), illustrating the challenge of defining the ground truth in transition areas.

| 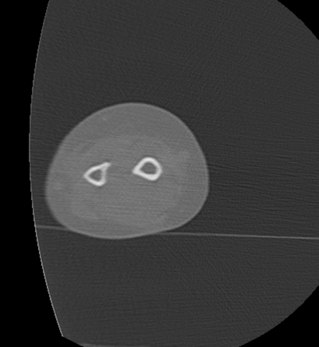  a: GT = forearm, MP = forearm | 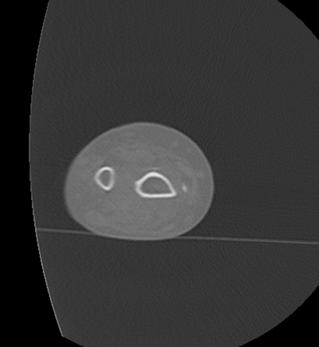  b: GT=forearm, MP = hand | 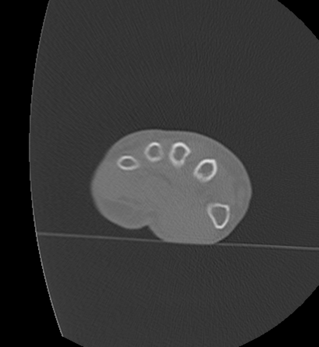  c: GT = hand, MP = hand |
| --- | --- | --- |

**Figure 7**: CT Upper extremity study. Images correctly inferred as forearm (a) and hand (c). The image in-between is misclassified as hand (b), illustrating the challenge of defining the ground truth in transition areas.

## Other Image Misclassifications

Definitions: GT = Ground Truth, MP = Model Prediction.

| 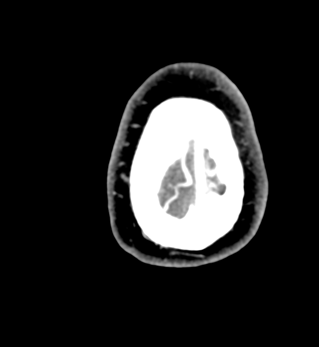  a: GT = head, MP = elbow | 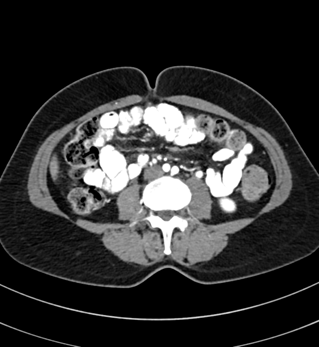  b: GT = pelvis, MP = hand | 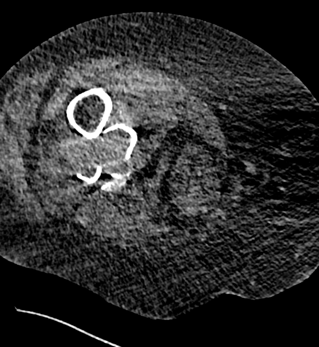  c: GT = knee, MP = shoulder |
| --- | --- | --- |
|  |  |  |

**Figure 8**: Examples of erroneous classifications (CT) prior to post-processing. Head image misclassified as elbow (a). This image of the top of the skull is not very specific and may mimick the overall configuration of bone and soft tissue seen in elbow images. Pelvis image misclassified as hand (b). It is possible that fecal tagging in the colon may have created some confusing imaging pattern. Knee image clasified as shoulder (c). Notice the combination of a bone fracture and poor image quality (large amount of noise). It is important to note that each of those examples are single occurences in their respective series. The post-processing step adequatly corrected these misclassifications using the anatomical predictions from neighbouring slices.

# Training Parameters

| **Parameters** | **CT Model** | **MRI model** |
| --- | --- | --- |
| Best Training epoch | 87 | 89 |
| Learning rate | 1e-05 | 1e-05 |
| Optimization algorithm | Nadam | Nadam |
| Batch size | 128 | 96 |
| Step size | 2948 | 865 |

**Table 3**: Training hyperparameters.

# Performance and Hardware Information

We used Google Cloud Platform to store data, manage our database of experiments, execute the experimentations, and run the inference engine. Docker containers were built with the necessary TensorFlow library and its dependencies. Training for each model took up to 24 hours on a dedicated cloud instance using Nvidia V100 GPU hardware. Inference through TensorFlow Serving on a dedicated cloud instance with Nvidia T4 can process more than 35,000 images per hour, which amount to roughly 4,000 series or 600 studies per hour. Everything was run on Linux through Python and node.js.
